# Clinical Significance of Preoperative Neutrophil – to - Lymphocyte Ratio in Renal Cell Carcinoma

**DOI:** 10.1590/S1677-5538.IBJU.2015.0397

**Published:** 2016

**Authors:** Alper Otunctemur, Murat Dursun, Huseyin Besiroglu, Kutan Ozer, Ozan Horsanali, Emin Ozbek

**Affiliations:** 1 Department of Urology, Okmeydani Training and Research Hospital, Istanbul, Turkey; 2 Department of Urology, Ataturk Training and Research Hospital, Izmir, Turkey

**Keywords:** Neutrophils, Lymphocytes, Carcinoma, Renal Cell, Epithelium

## Abstract

**Introduction:**

We investigated the prognostic significance of the neutrophil-to-lymphocyte ratio on tumor stage and Fuhrman nuclear grade in renal cell carcinoma.

**Methods:**

The records of 432 patients with RCC who underwent radical or partial nephrectomy between 2005 and 2014 were retrospectively reviewed. Patients were classified as group lower tumor stage(T1 + T2) and higher(T3 + T4). As like tumor stage, Fuhrman nuclear grade were classified lower (G1+G2) and higher(G3+G4) too. The best NLR cut off value was 3.01. Two sample t-test or Mann–Whitney U-test used for the continuous variables and a chi-square test or Fisher's exact test used for the categorical variables.

**Results:**

Among the 432 total patients analyzed in our study, there were 275 males (63.7%) and 157 females (36.3%). Mean laboratory values were CRP 2.73 ± 1.93 mg/dL (normal less than 0.3), neutrophil count 4,23 ± 1.46/μL, lymphocyte count 1,61 ± 0,61/μL and NLR 2.64 ± 1.24. According to our data, statistically pretreatment NLR significantly correlated with CRP (p<0.0001). And tumor patologic stage (p=0.08), tumor histologic grade (p<0.001) was significantly associated with NLR.

**Discussion:**

We compared the relationship of preoperative NLR and NC parameters with RCC tumor stage and grade. And NLR were found to have statistically significant higher T stage and grade at RCC. Further studies with more patients are needed to confirm our study.

## INTRODUCTION

Renal cell carcinoma (RCC) is the most common renal malignancy in adults and leads to a mortality rate of over 100.000 per year worldwide. In the United States renal cell carcinoma accounts for 2.3% of all cancer deaths (1). RCC is more prevalent in men than in women and occurs most often between 50-70 years of age. Cancer involving the renal parenchyma accounts for the majority of cases, while the minority of cases derive from the renal pelvis. The predominant subtype of RCC is a clear cell type that represents 80% of RCC, and is derived from the tubular epithelium (2). The incidence and the incidental detection of RCC in asymptomatic patients have been increasing worldwide (3-5). The increase can be partly explained by the widespread usage of ultrasound, abdominal computorized tomography (CT) and magnetic resonance imaging in recent years (6).

Cancer and inflammation are inextricably linked, and cancer patients have local and systemic changes in the inflammatory parameters. These include changes in peripheral blood cell numbers (neutrophils, lymphocytes, and neutrophil to the lymphocyte ratio [NLR]), phenotypes, and gene expression patterns, changes in the erythrocyte sedimentation rate, and alterations in the level of serum inflammatory cytokines, acute-phase proteins (C-reactive protein [CRP], fibrinogen, ferritin, albumin, and transferrin). CRP is a representative marker of a systemic inflammatory response and increased CRP is a poor prognostic factor in several cancer types, including RCC (7-9).

Another marker of systemic inflammatory response is NLR (10). It was recently reported that increased pretreatment NLR is associated with poor outcome in colorectal (11), gastric (12) and ovarian (13) cancer cases. To our knowledge, the prognostic value of NLR in RCC has not been investigated in similar studies. Therefore, the aim of this study was to evaluate the relationship between the tumor stage and grade which are commonly found in patients with renal cell carcinoma.

## MATERIALS AND METHODS

After receiving approval from our institutional review boards we retrospectively analyzed the prospective kidney cancer databases of the patients who underwent radical and partial nephrectomy between 2005 and 2014 at our institution. The databases contain information on the clinical presentation, demographics, comorbidity, pathological findings, and preoperative laboratory parameters of patients. A total of 432 patients were screened for the study. The variables abstracted from the databases included age, gender, various pathological parameters, lymphocyte, and neutrophil count. The clinical presentation was categorized as symptomatic or incidental. The tumors accompanied by pain, hematuria, abdominal mass, fever, or weight loss, were categorized as symptomatic tumors. The preoperative neutrophil - to - lymphocyte ratio (NLR) was calculated by dividing the neutrophil count (NC) by the lymphocyte count (LC).

The surgical specimens were processed according to standard pathological procedures and evaluated by the pathologists at our institution. The pathologic staging was performed using the 7th edition of the American Joint Committee on Cancer (AJCC). The histologic subtype was determined according to the 1997 World Health Organization Heidelberg classification, and the tumor nuclear grading was performed according to the Fuhrman nuclear grading system (14). There are several proposed cut off points to stratify LC, NC, and NLR (15-17). The best NLR cut off value was 3.01.

### Statistical analysis

The baseline characteristics of the subjects were compared using a two sample t-test or Mann–Whitney U-test for the continuous variables and a chi-square test or Fisher's exact test for the categorical variables. All the statistical tests were two-tailed, and the statistical significance was defined as P<0.05. All the analysis was conducted using SPSS version 15.0 (SPSS Inc., Chicago, Illinois, USA).

## RESULTS

Among the 432 total patients analyzed in our study, there were 275 males (63.7%) and 157 emales (36.3%). The demographic analyses and clinicopathologic characteristics are shown in Table-1. The median age at the time of surgery was 57.76±10.97. Radical and partial nephrectomy was performed in 379 (87.7%) and 53 (12.3%) patients, respectively. Of the tumors, 348 were incidental and 84 were symptomatic. The tumor pathologic stage was determined at a lower stage (T1 or T2) and at a higher stage (T3 or T4) in 360 (83.3%) and 72 (16.7%) of the patients respectively. Also, the histopatologic nuclear grades were stratified as lower grade (G1 or G2) and higher grade (G3 or G4) in 332 (76.9%) and 100 (23.1%) of the patients respectively (Table-1).

**Table 1:** Patient characteristics and pathological findings.

		No. Pts(%)
Average Age		57.76±10.97
**Gender**		
	Male	275 (63.7%)
	Female	157(36.3%)
**Presentation**		
	Incidental	348 (80.5%)
	Symptomatic	84(19.5%)
Radical Nephrectomy		379 (87.7%)
Partial Nephrectomy		53(12.3%)
**Stage**		
	Lower stage (T1-T2)	360 (83.3%)
	Higher Stage (T3-T4)	72(16.7%)
**Nuclear Grade**		
	Fuhrman Grade 1 and 2	332 (76.9%)
	Fuhrman Grade 3 and 4	100(23.1%)

Mean laboratory values were CRP 2.73±1.93mg/dL (normal less than 0.3), neutrophil count 4.23±1.46/μL, lymphocyte count 1.61±0.61/μL, and NLR 2.64±1.24. Patients were also stratified according to NLR using cutt off value 3.01. Of 360 patients with lower stage (T1 or T2), 146 were found to have NLR >3.01; while 41 patients with higher stage (T3 or T4) tumor have NLR >3.01. Additionally, of 100 patients with higher grade (G3 or G4) tumor, 71 were found to have NLR >3.01. The details were demonstrated in Table-2.

**Table 2:** The neutrophil/lymphocyte ratio according to tumor parameters and markers in surgery group.

Characteristic		NLR < 3.01	NLR > 3.01	P value
**Age**				0.486
	≥60	109	82	
	<60	136	105	
**Gender**				0.537
	Male	156	119	
	Female	89	68	
**CRP level**				0.015 *
	≥0.3	108	103	
	<0.3	137	84	
**Tumor Stage**				0.008 *
	T1+T2	214	146	
	T3+T4	31	41	
**Nuclear Grade**				<0.001 *
	G1+G2	216	116	
	G3+G4	29	71	698.4

* = Statiscally meaningful

According to our data, no statistically difference was found in NLR value in terms of age (≥60, <60) and gender distribution. The pretreatment NLR significantly correlated with CRP (p=0.015). The tumor patologic stage (p=008), and the tumor histologic grade (p<0.001) were significantly associated with NLR. The median NC was significantly associated with the tumor stage and the Fuhrman grade (p=0.048, p=0.021), but LC was not associated (p= 0.841, p=0.774). The detailes were depicted in Table-2 and 3.

**Table 3:** NC, LC and NLR associations with pathological parameters.

		Neutrophil Count			Lymphocyte Count	
		No. Pts	Median	P Value	Median	P Value
**Tumor Stage**				0.048 *		0.841
	T1+T2	360	4.08(3.1-6.2)		1.60(1.2-2.3)	
	T3+T4	72	5.01 (3.5-6.9)		1.71 (1.2-2.2)	
**Nuclear Grade**						
	G1+G2	332	3.96 (3.4-5.3)	0.021*	1.61 (1.3-2.1)	0.774
	G3+G4	100	5.11 (3.4-6.3)		1.67(1.3-2.2)	

* = Statiscally meaningful

## DISCUSSION

Many well-known prognostic factors exist, including the anatomical factors (the TNM stage and the tumour size), histological factors (the nuclear grade, the histological type, and the microscopic venous invasion), and clinical factors (symptoms, performance status, and anemia) (18). Molecular markers represented by carbonic anhydrase IX are also being investigated as potential prognostic factors for RCC, but the TNM stage and nuclear grade remain the most important prognostic factors (18, 19).

The neutrophil - to - lymphocyte ratio (NLR) is an easily measurable parameter of the systemic inflammation and stress in patients (10). Our study suggests that the pretreatment NLR may have some prognostic value for the renal cell carcinoma. In this retrospective study, the patients with the lower pretreatment NLR who underwent radical nephroctomy had a lower T stage and nuclear grade with the histopathologic results. This study shows that NLR is an independent prognostic factor after surgery for RCC.

Increasing evidence supports the involvement of systemic inflammation in cancer development and progression (20). A systemic inflammatory response can be assessed by the concentration of acute phase proteins, CRP, fibrinogen, ferritin, albumin and transferrin, or peripheral blood leukocyte components, including neutrophils and lymphocytes. CRP, which is an acute phase protein, has been widely studied as a prognostic parameter for various cancers (9, 21). Increased pretreatment CRP is associated with poor survival in patients with localized and metastatic RCC (7, 8). CRP kinetics also have an impact on the survival of patients with metastatic RCC since the decreased CRP during treatment is a predictor of a better prognosis (22). In regard to the prognostic significance of peripheral blood leukocyte components, neutrophilia and lymphocytopenia were reportedly associated with a poor prognosis in patients with metastatic RCC (23, 24). However, the prognostic significance of the peripheral blood leukocyte component in patients with localized RCC remains unclear.

A high NLR reflects an increased neutrophil and/or a decreased leucocyte ratio. It is generally accepted that the inflammatory processes in the tumor microenvironment play a crucial role in promoting proliferation, invasion, and metastasis of the malignant cells (20, 25). The infiltrating leucocytes, including neutrophils and lymphocytes, are important factors in this process (20). Neutrophilia has been associated with malignancy. However, the cause is not completely understood. Neutrophils in the peripheral blood, or in the tumor microenvironment, were shown to produce pro-angiogenic factors including the vascular endothelial growth factor to stimulate tumor development and progression (26). The cytokines involved in cancer-related inflammation, including interleukin-6 (IL-6) and tumor necrosis factor- alpha (TNFα), may induce neutrophilia (27, 28). The para-neoplastic production of myeloid growth factors by the cancer cells may represent an additional cause of neutrophilia (29). Hence, a high peripheral neutrophil level may indicate a cancer-related inflammation or tumor progression, and predict a poor clinical outcome. The immune cells that infiltrate into or around the tumor engage in dynamic and extensive crosstalk with the cancer cells (30).

Over the past decade, there has been growing evidence that lymphocytes operate as crucial components for the adaptive immune system, and are the cellular basis of cancer immunosurveillance and immunoediting (31). Furthermore, infiltrating lymphocytes have been reported to indicate the generation of an effective anti-tumor cellular immune response (32). Therefore, a low lymphocyte count may be responsible for an inadequate immunologic reaction to the tumor, and consequently a weakened defence against cancer, resulting in poor prognosis (33). Activated specific CD8+T cells were shown to control tumor growth by cytotoxic activity and inducing the apoptosis of the tumor cells (34). CD4+T cells are crucial for screening cytokines such as IL-2, which are essential for CD8+T cell growth and proliferation. Furthermore, recent reports reveal that the activation of CD4+T cells is required for the immunization of the CD8+T cells against cancer (35). In vitro studies showed that the cytolytic activity of lymphocytes and natural killer cells were suppressed when co-cultured with neutrophils, and the extent of the suppression was proportionally enhanced by the addition of neutrophils (36, 37). Accordingly, an elevated pre-treatment NLR was reported to correlate with the reduced survival in several types of cancers.

In the RCC field, the first report addressing the usefulness of NLR as a prognostic indicator was published by Ohno et al. and focused on localized RCC (15). In published studies to date, only patients with RCC (38, 39) or the subtypes were the predominantly clear cells (16, 40) included. In a multivariable model, a variable that combined a categorically coded pretreatment and posttreatment NLR, attained statistical significance. Pichler et al. validated preoperative NLR as an independent prognostic factor in 678 patients with nonmetastatic clear cell RCC (38). In a prospective study, in 83 patients categorically coded ANC and ALC were not associated with disease-free survival, but this study accrued relatively few patients (16).

This study had some deficiencies. Firstly, it is a retrospective study with the limited number of patients included. It is unavoidable to state that the post follow-up period is as important as the pathology in the follow-up of the patients involved in the study and the aggressiveness of the tumor. Secondly, our results were based on the experience of a single institution in Turkey with a <600 patients with RCC, and there are substantial differences in RCC incidence and mortality rates between Western countries and Turkey. Therefore, the relationship between RCC and NLR should be validated through massive studies worldwide. The factors that are definitely attested to be effective on RCC risk (such as smoking, obesity, hypertension, genetic susceptibility, and enviroment etc.) were not included in the study. Moreover, although there still is no agreement as to which grading system should be used, the most common system is the one proposed by Fuhrman et al. (14). The Fuhrman grade is based on the nuclear size and shape and the prominence of nucleoli, and we know that when the surgical specimens are evaluated by different pathologist there can be some differences. Thus, it would be better to work with one single pathologist.

Although it is debatable whether or not to estimate the progression of RCC based on the preoperative blood parameters; our findings are valuable as they show the value of NLR in this respect.

## CONCLUSIONS

In conclusion, we have found that patients with higher T stage and grade renal cell carcinoma have elevated level of NLR which provides a positive link between RCC and inflammation. This is a practical assessment tool that can easily be used for the risk stratification and prognostic models. Further studies with more patients are needed to confirm our study findings.
